# Attention Patterns in Children with Attention Deficit Disorder with or without Hyperactivity

**DOI:** 10.1100/tsw.2003.94

**Published:** 2003-11-13

**Authors:** Gil Zalsman, Orat Pumeranz, Gabriel Peretz, David H. Ben-Dor, Sharon Dekel, Neta Horesh, Tsvi Fischel, Eitan Nahshoni, Pablo H. Goldberg, Jonathan Sever, Alan Apter

**Affiliations:** Adolescent Inpatient Unit, Geha Psychiatric Hospital, Petah Tikva, Israel

**Keywords:** Continuous Performance Test, CPT, attention deficit disorder with or without hyperactivity, ADHD, ADD, methylphenidate, Israel

## Abstract

The objective of this study was to differentiate the attention patterns associated with attention deficit disorder with or without hyperactivity using continuous performance test (CPT). The diagnoses were based on the DSM-III, III-R, and IV criteria and of the 39 children who participated in the study, 14 had attention deficit disorder with hyperactivity (ADDH) and 11 had attention deficit disorder without hyperactivity (ADDWO), while 14 normal children served as a control group. Attention patterns were examined according to the performance of subjects on the CPT and parental scores on the ADHD Rating Scale, the Child Attention Profile, and the Conners Rating Scale. CPT performances were assessed before and after administration of 10 mg methylphenidate. We found as hypothesized that the CPT differentiated between the ADDH and ADDWO groups. However, contrary to our expectations, the ADDH children made more omission errors than the ADDWO children; they also showed more hyperactivity and impulsivity. The performance of both groups improved to an equal degree after the administration of methylphenidate. It is conluded that different subtypes of the attention deficit disorders are characterized by different attention profiles and that methylphenidate improves scores on test of continuous performance.

### INTRODUCTION

The clinical picture of attention deficit, excessive motor activity, and impulsivity is a common finding among children referred to Mental Health Clinics. Nevertheless, despite the extensive research in this area, of attention deficit disorder remain unclear. Some definitions of attention deficit disorder focus on hyperactivity as the central factor, and others, like that of the DSM-III, emphasize on the attention deficit and impulsivity.

According to factor analyses, the symptoms of attention deficit disorder reflect two independent dimensions: (1) hyperactivity and impulsivity, and (2) attention deficit, disorganization, and difficulty in completing tasks. On cluster analyses, these dimensions significantly matched the independent DSM-III diagnoses of attention deficit disorder with hyperactivity (ADDH) and attention deficit disorder without hyperactivity (ADDWO)[1]. Furthermore, there is clinical evidence that many children referred to health services because of developmental problems, especially in academic and social fields, suffer from attention deficit but not from hyperactivity. This is supported by findings of significant differences not only in the main symptomatology of attention deficit disorder, but also in cognitive behavioral, emotional, and family patterns[2].

Barkley[3,4] found that although the salient symptom of both types of attention deficit disorder is short attention span — as reported by teachers and mothers and as documented in clinical reports[2] — the specific attention difficulties vary. Children with ADDH show behavioral disorganization and disinhibition, i.e., difficulties in preserving attention, whereas children with ADDWO show lower cognition and inward-directed attention, i.e., difficulties in focusing attention[3,5,6]. ADDH children have been found to be nervous, unreliable, reckless, distractible, and easily fatigued, and to work in a disorganized manner, while ADDWO children are confused, dreamy, indifferent, lazy, and slow, and show poor motivation and difficulties in completing a task[4,7,8]. Patients with ADDWO performed worse than those with ADDH on the coding subtest of the Wechsler Intelligence Scale for Children - Revised. They also had poorer consistent recall of verbal material from memory, but fewer problems with behavior unrelated to the task when the task was long and alertness was needed[4]. In their examination of the hyperactivity-impulsivity factor, Lahey et al.[1] reported that both attention deficit disorder groups showed more restlessness and frustration than normal children, but the symptoms of the children with ADDH were related directly to impulsivity , and of the children with ADDWO, to disorganization. Thus, children with ADDH act before they think and often change activity, and children with ADDWO often lose things, need more mentoring, and seem more inattentive than children with ADDH. As for the hyperactivity factor, compared to children with ADDWO, children with ADDH are more hyperactive[2] and more talkative, and they have a greater tendency to engage in dangerous activity[9].

These differences in hyperactivity- impulsivity between the attention deficit disorder subgroups point to a difference in their related behavioral-emotional profiles. Children with ADDH are aggressive and have behavioral problems, whereas children with ADDWO are shy and have anxiety problems[10]. The first group has been found to lack feelings of guilt, and the second suffers from depression[11]. Though children of both groups are rated as "less wanted" by their peers[12], ADDH children are less popular than ADDWO children[13]. Lahey and Carlson[10] also found that the pattern of undesirability is different between the groups: ADDH children are socially rejected whereas ADDWO children withdraw themselves from their peers. Both groups also show worse neurocognitive functioning than normal children[14], but ADDH children have more difficulty than ADDWO children in changing strategy according to the task[15], and they show more neurological abnormalities[14]. ADDH children have more problems with speech, whereas ADDWO children have more problems with language[2].

Family quarrels and a tendency to psychiatric disorders among mothers are more common in the ADDH group[2]. Relatives of ADDH children have a higher rate of attention deficit disorder and substance abuse than those of normal children, while relatives of ADDWO children have more anxiety and learning disorders. There are also differences in medical history. Early developmental evaluation shows ADDH children to be more active and stubborn, and ADDWO children to have low motor coordination[4]. In addition, ADDH appears to be a consistent disorder which often evolves into a behavioral disorder, whereas ADDWO is unstable over long periods and frequently evolves into an emotional disorder[2]. Differences in response to medication are less clear. Cantwell and Baker[2] found no difference in the effect of Ritalin between the attention deficit disorder groups, though others[3,4] reported that ADDWO children react better to low doses of Ritalin and ADDH children react better to high doses.

In summary, much of the literature points to distinct disorders of attention deficit, with the difference not limited to the demonstration of hyperactivity symptoms. A few empirical studies have been conducted on these differences, but they were limited by methodological problems: small number of subjects, lack of control of sex and age variables, and unreliable measurements based on evaluations of teachers and parents[10], which did not differentiate attention problems from other overt behavioral problems and placed too much stress on the identification of behavioral problems and hyperactivity[16]. The present study was prompted by the need for a reliable tool to better identify the differences between ADDH and ADDWO. The DSM-III-R and DSM-IV were released after we started our study, so the results were expanded to include the division of the study groups by these diagnostic categories as well.

The Test of Variables of Attention (TOVA), a computerized continuous performance test, has been found to be effective in differentiating between children with attention deficit hyperactivity disorder (ADHD) (DSM-III-R definition) and normal children[16]. In this study, we examined the reliability of the TOVA in differentiating children with ADDH from children with ADDWO. We hypothesized that the TOVA would be susceptive to the formation of specific profiles of attention deficit according to the subcategories defined by the DSM-III. We assumed that attention variables loaded with impulsivity and heterogenic reactions would be more characteristic of the difficulties of children with ADDH, and variables loaded with confusion and slowness would more accurately characterize children with ADDWO. For further confirmation, the outcome of the TOVA was matched with parental scores on the ADHD Rating Scale, Child Attention Profile (CAP), and Conners Rating Scale. We further hypothesized that the ADDH group would have lower overall scores than the ADDWO group on these questionnaires, with no intergroup differences in attention, but higher scores for hyperactivity- impulsivity for the ADDH children. Finally, in light of the current debate on the influence of methylphenidate (Ritalin) on attention disorders, we retested the children after administration of the drug. We hypothesized that both groups would show improvement with methylphenidate, but the treatment would be more effective in the ADDH group.

### METHODS

#### Subjects

The study population included three groups of Israeli children matched for sex, age, socioeconomic status, and intelligence (normal range). The children with attention deficit were selected from among 36 children referred to a university-affiliated hospital for attention and behavioral problems; 11 were excluded because of comorbidity of neurological, motor, hearing, or vision disorders, or mental retardation or psychosis. Of the remainder, 14 children (13 males, 1 female) had ADDH and 11 (9 males, 2 females) had ADDWO. The third group consisted of 14 normal children (10 males, 4 females) recruited from the community. Mean ages of the groups were 10.5 years (range 6–12 years), 10.2 years (range 8–13 years), and 11 years (range 9–13 years), respectively.

#### Procedure

The diagnoses of ADDH and ADDWO were made by senior child psychiatrists and based on the DSM-III criteria. However, we were able retrospectively to reclassify the children using the DMS-III-R and DSM-IV. This yielded, for the DSM-III-R, 24 children with ADHD and for the DSM-IV, 11 children with

ADHD-inattentive type (ADHD-I), 4 with ADHD hyperactive-impulsive type (ADHD-H), and 10 with combined type (ADHD-CT). Trained technicians administered the TOVA individually during the morning hours. Parents were asked to complete three related questionnaires. On completing the TOVA, the children in the study groups were given 10 mg methylphenidate, after which half of them repeated the test.

### Measures

The TOVA (Minnesota Computer Assessment)[16] is a computerized visual continuous performance test. The duration of the test is 22.5 min, and its completion does not require language or the ability to differentiate left from right. The effect of training is minor. The subject is seated facing a screen, and one of two visual stimuli is shown every 2 sec for 100 thousandth of a second: either a small square near the top edge of the screen or a small square near the bottom edge. The subject is instructed to press a bar as quickly as possible if the first stimulus is presented and to do nothing if the second is presented. Four main performance dimensions are measured: (1) omission error — not responding to the target stimulus (demonstrates attention deficit), (2) commission error — responding to the control stimulus (demonstrates impulsivity), (3) response time — mean time to correct response to the target stimulus, and (4) complete response time variance — standard deviation of the mean time to correct responses to the target stimulus (demonstrates attention preservation). A t-score of 65 or more in one of these dimensions is considered pathological[16], and a deviation from the norm in even one dimension is enough for a diagnosis of attention deficit disorder. In addition, there are two minor dimensions: (1) anticipatory response — responding after less than 200 thousandths of a second after presentation of the stimulus, i.e., before it is possible to differentiate which stimulus is presented (demonstrates impulsivity); and (2) postcommission response — slow time to a correct response after an incorrect one (demonstrates impulsivity).

The parents completed the Hebrew version of the following three questionnaires:

- The Child Attention Profile (CAP)[17] evaluates the presence of an attention problem and/or hyperactivity. The questionnaire is based on the Child Behavior Checklist-Teacher Report Form[13] and includes 12 items rated on a scale of 0 to 2. A score above the norm indicates a clinical problem. In our study, parents were asked to refer to the previous week when completing the questionnaire.
- The ADHD Rating Scale[4] evaluates the presence of ADHD symptoms in children. The questionnaire is based on the DSM-III-R criteria and includes 14 items rated on a four-point scale, which examine two factors: attention deficit-hyperactivity and impulsivity -hyperactivity. A score of 1.5 deviations above the mean indicates a clinical problem in each factor.
- The Conners Rating Scale for Parents and Teachers[18] includes 10 items rated on a four-point scale. The questionnaire is based on the Conners Teacher Rating Scale[19] which examines two factors: attention deficit and hyperactivity-aggressiveness. The test-retest reliability and internal consistency are high[18].

### Data Analysis

The data were analyzed with one-tailed MANOVA and Scheffe's pairwise analysis.

## RESULTS

### TOVA — Initial Testing

Table 1 presents the mean scores and standard deviations for the TOVA. Significant between-group differences in the four main (T) scores found by MANOVA were tested by ANOVA. To examine the differences in the pathological dimensions between the study groups, the same data analysis was conducted as for the main scores, with each dimension with a pathological score (65 points or more) assigned a value of 2, and each dimension with a normal score assigned a value of 1; the dimensions receiving a value of 2 were then summed. In addition, the mean of the four T scores was measured. Finally, we compared the groups for the main diagnosis of the TOVA, i.e., presence or absence of attention deficit disorder.

**TABLE 1**
**Mean Scores and Standard Deviations of the TOVA According to Diagnostic Method**

| | | | DSM-III | | | | DSM-III-R | | DSM-IV | | | | | |
|---|---|---|---|---|---|---|---|---|---|---|---|---|---|---|
| | Control | | ADDH | | ADDWO | | ADHD | | ADHD-I | | ADHD-H | | ADHD-CT | |
| | M | SD | M | SD | M | SD | M | SD | M | SD | M | SD | M | SD |
| **Main score (T)** | | | | | | | | | | | | | | |
| Omission errors | 48.5 | 3.5 | 57.9 | 14.6 | 56.1 | 18.2 | 57.5 | 16.2 | 58.6 | 18.05 | 35.9 | 6.3 | 56.8 | 17.3 |
| Commission errors | 48.1 | 4.4 | 52.8 | 10.7 | 45.7 | 5.6 | 50.1 | 4.6 | 47.06 | 6.3 | 50.9 | 12.8 | 52.2 | 11.03 |
| Response time | 52.9 | 8.8 | 61.2 | 16.0 | 68.8 | 10.3 | 64.4 | 14.3 | 63.6 | 9.1 | 54.7 | 8.3 | 67.2 | 18.9 |
| Response time var. | 47.9 | 8.0 | 68.9 | 17.2 | 60.7 | 14.1 | 5.3 | 16.5 | 60.9 | 14.1 | 67.9 | 16.0 | 69.0 | 18.6 |
| **Elaborated score** | | | | | | | | | | | | | | |
| Pathol. dimen. | 0.26 | 0.07 | 1.21 | 0.97 | 1.09 | 0.53 | 1.16 | 0.81 | 1.09 | 0.5 | 0.7 | 0.5 | 1.4 | 1.07 |
| Test diagnosis | 1.07 | 0.26 | 1.8 | 0.36 | 1.9 | 0.3 | 1.87 | 0.33 | 1.9 | 0.3 | 1.7 | 0.5 | 1.9 | 0.3 |
| Mean scores | 49.4 | 4.1 | 60.2 | 9.3 | 57.8 | 4.4 | 69.3 | 7.6 | 58.1 | 4.4 | 56.9 | 7.3 | 61.3 | 0.1 |
| CPTOT1 | 21.9 | 8.2 | 53.6 | 33.9 | 45.2 | 49.4 | 51.16 | 41.3 | 55.4 | 52.9 | 40.8 | 34.9 | 47.4 | 9.6 |
| **Summed data** | | | | | | | | | | | | | | |
| Omission errors | 2.7 | 3.8 | 20.8 | 21.1 | 12.36 | 15.7 | 17.6 | 19.2 | 19.4 | 20.9 | 12.1 | 15.1 | 16.4 | 19.6 |
| Commission errors | 19.2 | 6.1 | 32.8 | 17.1 | 32.8 | 39.5 | 33.5 | 28.8 | 36.0 | 39.6 | 28.5 | 20.5 | 31.0 | 15.8 |
| Response time | 431.1 | 67.9 | 487.9 | 96.7 | 512.1 | 92.3 | 499.1 | 95.6 | 511.0 | 86.3 | 410.0 | 53.5 | 520.3 | 99.7 |
| Response time var. | 117.1 | 34.2 | 208.5 | 72.2 | 184.5 | 42.7 | 198.7 | 62.3 | 196.3 | 52.2 | 185.0 | 63.5 | 205.0 | 74.1 |
| Postcom. time | 458.9 | 100.6 | 526.8 | 134.7 | 522.6 | 71.5 | 461.9 | 97.6 | 515.6 | 68.8 | 418.0 | 98.4 | 578.0 | 122.9 |
| Postcom. var. | 123.5 | 51.2 | 229.5 | 107.0 | 187.4 | 121.5 | 125.6 | 50.1 | 204.1 | 120.8 | 184.0 | 36.5 | 227.9 | 129.5 |

TOVA = Test of Variables of Attention; ADDH = attention deficit disorder with hyperactivity; ADDWO = attention deficit disorder without hyperactivity; ADHD = attention deficit hyperactivity disorder; I = inattentive type; H = hyperactive-impulsive type; CT = combined type; CPTOT1 = sum of omission and commission errors; Pathol. dimen. = pathological dimensions; Postcom. = postcommission; var. = variance.

We found that the number of pathological dimensions, the test diagnosis, and the mean T score significantly differentiated the control group from the study groups. Nonsignificant differences were found between the two study groups.

### Omission Errors

The ADDH group received the highest score, followed by the ADDWO and the control groups, but the differences were not significant. Division of the patients by the DSM-III-R criteria yielded a significant difference between the ADHD group and the controls (DSM-III-R) (F = 4.32, $p < 0.05$). When the patients were reclassified according to the DSM-IV criteria, the order of scoring was: ADHD-I, ADHD-CT, ADHD-H, and controls; none of these differences was significant.

Regarding total number of errors, the ADDH group scored significantly higher than the control group and the controls ($p < 0.02$). Significance was also reached between the ADHD group (DSM-III-R) and the controls ($p < 0.01$). Division of the test showed that the ADDH group made significantly more errors than the controls in the first half of the test (F = 4.6, $p < 0.02$) and the ADHD group did so in both halves ($p < 0.01$ and $p < 0.01$, respectively). Analysis of the omission error score for pathology yielded a significant difference only according to the DSM-III-R diagnostic criteria (F = 4.52, $p < 0.05$).

### Commission Errors

The ADDH group had significantly higher scores than the ADDWO group (F = 2.95, $p < 0.05$). The ADHD group also scored higher than the control group. When DSM-IV categories were used, the ADHD-CT group scored highest, followed by the ADHD-H group, the control group, and the ADHD-I group; none of these differences was significant.

The number of errors made significantly differentiated the ADDH group from the control group (F = 2.54, $p < 0.05$), and the ADHD group from the control group (F = 5.33, $p < 0.02$). Analysis of the dimensions with pathological scores yielded no significant between-group differences. The sum of the omission and commission errors (CPTOT1) was significantly higher for the ADDH and ADHD groups than for the control group (F = 3.28, $p < 0.05$ and F = 7.34, $p < 0.01$, respectively). The mean T score derived from both errors (CPTOT2) was significant only when the DSM-III-R criteria were used (F = 4.11, $p < 0.02$).

### Response Time

The T score differentiated between the study groups and the control group irrespective of which DSM criteria were used to make the diagnosis. Although the response time of the ADDWO group was higher than that of the ADDH group, differences were significant only in comparison to the control group (F = 5.18, $p < 0.01$). The difference between the ADDH group and controls was also significant (F = 6.29, $p < 0.02$). Findings according to the DSM-IV were as follows (high to low): ADHD-CT, ADHD-I, ADHD-H, controls; findings were significant only for the ADHD-CT group vs. the control group (F = 3.68, $p < 0.05$).

Regarding general response time (summed data), significant differences were found between each of the DSM-III groups and the controls ($p < 0.01$ for ADHD and $p < 0.05$ for ADDWO). There was also a significant difference between the ADHD-CT group (DSM-IV) and the control group (F = 3.94, $p < 0.02$). Division of the test yielded significant differences between the ADDWO and control groups for the second half (F = 3.18, $p < 0.05$), the ADDH-CT and ADDH-H groups and the control group for the first half (F = 3.24, $p < 0.05$), and the ADDH-CT group and the control group for the second half ($p < 0.05$). The response time of the ADHD group was significantly lower than that of the control group in both halves ($p < 0.05$ and $p < 0.05$). The dimensions with pathological scores differentiated only between the ADDWO group and the control group (F = 5.18, $p < 0.01$). According to the DSM-III-R criteria, the pathological score significantly differentiated between the ADHD group and the control group (F = 4.69, $p < 0.05$). The ADHD-I group showed significantly greater pathology than both the control and the ADHD-H groups (F = 5.25, $p < 0.01$).

#### *Response Time Variance*

The T score was significantly higher for the ADDH group than for the control group, though a nonsignificant difference was noted between the two disordered DSM-III groups. The ADHD group scored significantly higher than the control group (F = 12.18, $p < 0.002$). According to the DSM-IV criteria, the ADHD-CT and ADHD-H groups scored significantly higher than the controls (F = 5.3, $p < 0.01$). Regarding mean response time variance, the ADHD group (DSM-III-R) and ADHD-CT group (DSM-IV) scored significantly higher than the controls in both total score (F = 6.7, $p < 0.001$) and second half of the test (F = 6.28, $p < 0.01$). Analysis of the score deviation from the norm yielded a significant difference between the ADDH group and the controls (F = 5.5, $p < 0.01$), between the ADHD-H group and both the ADHD-I and control groups (DSM-IV), and the ADHD-CT group and the control group (F = 5.29, $p < 0.01$). The ADHD group had significantly greater pathology than the control group (F = 12.04, $p > 0.002$).

#### *Postcommission Response*

To measure this variable, we evaluated the response time after commission errors and the variance in the response time. The response time variance was significantly smaller for the ADDH group compared to the control group (F = 4.25, $p < 0.05$) and for the ADHD-CT group compared to the ADHD-H group (F = 3.8, $p < 0.05$) (but not to controls). The variance was significantly larger for the ADHD-H group than for the control group (F =4.25, $p < 0.05$).

#### *Anticipatory Response*

This did not distinguish between the groups by any of the diagnostic criteria.

#### *Effect of Methylphenidate*

The analysis of variance of the retest scores included the main scores, elaborated scores, and other data derived from the TOVA. As shown in Table 2, methylphenidate significantly improved retest performance, but no significant difference was found between the disordered groups (DSM-III only).

### Parental Questionnaires

All three questionnaires were found to be valid for our population: CAP–Cronbach's alpha = 0.92, Spearman-Brown split test = 0.97; ADHD Rating Scale–Cronbach's alpha = 0.95, Spearman-Brown = 0.96; Conners Scale–Cronbachs' alpha = 0.90, Spearman-Brown = 0.87. Correlation analysis between the questionnaire scores and the diagnoses according to the DSM-III, -III-R, and -IV yielded several findings.

#### *CAP*

Two measures were analyzed: sum of general answers and sum of answers by attention and hyperactivity factors. The general diagnosis significantly correlated with the DSM-III (r = 0.37, $p < 0.05$), the DSM-III-R (r = 0.57, $p < 0.01$), and the DSM-IV (r = 0.57, $p < 0.01$), and the factor diagnosis significantly correlated with the DSM-III (r = 0.55, $p < 0.01$) and DSM-III-R (r = 0.56, $p < 0.01$).

**TABLE 2**
**Mean Scores and Standard Deviations of TOVA Before and After Methylphenidate (MPH)**

| | Before MPH | | After MPH | | Significance | |
|---|---|---|---|---|---|---|
| | M | SD | M | SD | F | *p* |
| **Main score** | | | | | | |
| Omission errors | 57.4 | 16.2 | 47.5 | 8.7 | 7.35 | 0.02 |
| Commission errors | 49.9 | 9.5 | 46.08 | 11.9 | N.S. | |
| Reaction time | 64.79 | 14.3 | 58.1 | 15.3 | 8.68 | 0.007 |
| Reaction time variance | 64.6 | 16.2 | 50.2 | 12.2 | 15.67 | 0.001 |
| **Elaborated score** | | | | | | |
| Test diagnosis | 1.88 | 0.3 | 1.4 | 0.5 | 19.76 | 0.001 |
| Pathological dimensions | 1.16 | 0.8 | 0.4 | 0.6 | 19.01 | 0.001 |
| Mean score | 59.2 | 7.6 | 50.4 | 7.5 | 19.76 | 0.001 |
| CPTOT1 | 49.8 | 41.0 | 27.5 | 26.4 | 7.5 | 0.02 |
| CPTOT2 | 53.7 | 10.7 | 46.8 | 8.3 | 7.6 | 0.02 |
| **Summed data** | | | | | | |
| Omission errors | 17.8 | 19.04 | 6.6 | 9.5 | 11.5 | 0.002 |
| Commission errors | 32.8 | 28.4 | 20.9 | 19.6 | N.S. | |
| Reaction time | 498.5 | 93.6 | 474.5 | 111.0 | N.S. | |
| Reaction time variance | 198.0 | 61.1 | 138.1 | 54.3 | 12.9 | 0.001 |

#### *ADHD Rating Scale*

Three measures were analyzed: sum of answers, summing of answers by impulsivity and attention factors, and sum of symptoms. The answer sum diagnosis significantly correlated with the DSM-III-R (r = 0.51, $p < 0.05$) and DSM-IV (r = 0.60, $p < 0.01$), and the symptom sum diagnosis with the DSM-III (r = 0.48, $p < 0.01$), DSM-III-R (r = 0.78, $p < 0.01$), and DSM-IV (r = 0.79, $p < 0.01$). The factor diagnosis significantly correlated with the DSM-III-R (r = 0.49, $p < 0.05$) and DSM-IV (r = 0.65, $p < 0.91$).

#### *Conners Scale*

A significant correlation was found between the sum of answers diagnosis (>15 considered pathological) and the DSM-III-R (r = 0.57, $p < 0.01$) and DSM-IV (r = 0.57, $p < 0.01$).

### Differences in Scores between the Groups

Finally, MANOVA was conducted to examine differences in scores between the groups. When differences were significant, ANOVA was performed with language tests to determine the direction of the difference (see Table 3).

**TABLE 3**
**Mean Scores and Standard Deviations on the Questionnaire According to Diagnostic Methods**

| | | | DSM-III | | | | DSM-III-R | | DSM-IV | | | | | |
|---|---|---|---|---|---|---|---|---|---|---|---|---|---|---|
| | Control | | ADDH | | ADDWO | | ADHD | | ADHD-I | | ADHD-H | | ADHD-CT | |
| | M | SD | M | SD | M | SD | M | SD | M | SD | M | SD | M | SD |
| **CAP** | | | | | | | | | | | | | | |
| Answer sum | 3.07 | 4.36 | 16.9 | 2.9 | 13.4 | 5.1 | 15.8 | 3.9 | 13.4 | 5.1 | 17.2 | 0.9 | 16.8 | 3.4 |
| Attention sum | 2.1 | 2.84 | 9.8 | 1.9 | 9.8 | 3.5 | 10.0 | 2.5 | 9.5 | 3.3 | 9.2 | 1.5 | 10.4 | 2.3 |
| Hyperactivity sum | 1.0 | 1.73 | 7.07 | 1.9 | 3.6 | 2.3 | 5.8 | 2.4 | 3.9 | 2.6 | 8.0 | 0.8 | 6.4 | 2.1 |
| **ADHD Rating Scale** | | | | | | | | | | | | | | |
| Answer sum | 4.2 | 5.3 | 27.8 | 6.6 | 21.1 | 7.9 | 25.5 | 7.4 | 21.0 | 7.9 | 26.5 | 4.7 | 28.4 | 7.3 |
| Attention sum | 2.8 | 3.6 | 17.4 | 4.5 | 14.8 | 5.9 | 16.7 | 5.1 | 14.2 | 5.7 | 16.2 | 2.2 | 18.5 | 5.3 |
| Impulsiveness sum | 2.3 | 3.01 | 16.0 | 3.8 | 9.8 | 4.1 | 13.7 | 4.7 | 10.2 | 4.3 | 16.5 | 3.6 | 15.4 | 4.4 |
| Symptom sum | 0.6 | 1.44 | 10.4 | 2.8 | 6.7 | 4.1 | 9.1 | 3.5 | 6.6 | 4.1 | 10.0 | 2.8 | 10.6 | 2.9 |
| **Conner Scale** | | | | | | | | | | | | | | |
| Answer sum | 3.6 | 4.3 | 16.8 | 4.9 | 12.5 | 5.1 | 13.4 | 5.2 | 12.3 | 5.6 | 19.2 | 21.8 | 16.0 | 5.2 |

ADDH = attention deficit disorder with hyperactivity; ADDWO = attention deficit disorder without hyperactivity; ADHD = attention deficit hyperactivity disorder; I = inattentive type; H = hyperactive-impulsive type; CT = combined type.

### *CAP*

Significantly higher answer sum scores were noted for the ADDWO and ADDH groups compared to controls; the scores of the ADDH group were also significantly higher than those of the ADDWO group. This was also true for the DSM-IV disordered groups compared to the controls (F = 25.7, $p < 0.00001$). The DSM-III and DSM-IV disordered groups had significantly higher scores than controls for the factor sum and a significantly lower score than the ADDH group in hyperactivity (F = 32.44, $p < 0.00001$). The ADHD-I group and the control group scored significantly lower than the ADHD-H group and the ADHD-CT group (F = 18.32, $p < 0.00001$) in the hyperactivity factor sum.

### *ADHD Rating Scale*

Significantly higher scores for the sum of the answers were noted for both the ADDWO and the ADDH groups compared to controls (F = 44.8, $p < 0.00001$). Furthermore, the ADDH group scored significantly higher than the ADDWO group. For the DSM-IV categories, the disordered groups score significantly higher than controls, with the ADHD-CT patients scoring significantly higher than the ADHD-I patients (F = 29.6, $p < 0.00001$). Regarding the factor sum score, both the DSM-III and DSM-IV disordered groups scored significantly higher than the control group in attention (F = 34.63, $p < 0.00001$ and F = 24.8, $p < 0.00001$) and the ADDWO group scored significantly higher than the control group, and significantly lower than the ADDH group on impulsivity (F = 48.18, $p < 0.00001$). All DSM-IV disordered groups scored significantly higher than the controls, with the ADHD-H and ADHD-CT children showing a significantly higher score than the ADHD-I children (F = 27.4, $p < 0.00001$). Finally, the ADDWO group had significantly more symptoms than the controls and significantly fewer symptoms than the ADDH group (F = 37.2, $p < 0.0001$). All DSM-IV disordered groups had significantly more symptoms than the control group. In addition, the ADHD-I group had significantly fewer symptoms than the other two disordered groups (F = 25.23, $p < 0.00001$). For all three measures, the ADHD group scored significantly higher than the control group.

#### Conners Scale

Scores were significantly higher for the DSM-III disordered groups than the control group, and for the ADDH group than the ADDWO group (F = 24.5, $p < 0.0001$). The ADHD group was evaluated as being significantly more disordered than the control group (F = 50.2, $p < 0.00001$). Division of the patients according to the DSM-IV criteria yielded significantly higher scores for the disordered groups compared to the control group and for the ADHD-H group compared to the ADHD-I group (F = 17.51, $p < 0.00001$).

### Correlation between TOVA and Questionnaire Scores

#### Omission Error

No significant positive correlation was found between the TOVA T scores and the questionnaire scores, although there was a correlation between the sum of omission errors and all the evaluation questionnaire scores and between the sum of omission errors and both the answer sum and attention factor scores of the CAP.

#### Commission Error

There was no correlation between the TOVA T scores or the sum of errors score with the questionnaire scores.

#### Reaction Time

The T score significantly and positively correlated with the answer sum score of the evaluation questionnaire and with the attention factor score of the evaluation questionnaire and of the CAP. The total reaction time also correlated significantly and positively with all the evaluation questionnaire scores, and with the attention factor of the CAP.

The T score and sum of reaction time variance correlated significantly and positively with all three questionnaire scores. The mean T score correlated significantly and positively with all the questionnaire scores. The pathological dimensions score correlated significantly and positively with all the questionnaire scores. The CPTOT1 score correlated significantly and positively with the attention factor of the CAP.

### DISCUSSION

This study attempted to identify empirically the attention patterns of children with ADDH and ADDWO using the TOVA. Although the literature discusses the major differences between these groups, the differentiation of their attention patterns has not been extensively evaluated. The present report supports the notion that attention deficit disorder is a two-dimensional entity. The ADDH patients were more impulsive than the ADDWO group and had more difficulties concentrating and focusing, while the ADDWO group was slower and had more difficulties maintaining attention. The ADDH group also scored lower on CAP and Conners Scale than the ADDWO group, and a significant correlation was found between the questionnaires and the TOVA scores. The TOVA was susceptive to the improvement in performance with methylphenidate.

Our findings are in accordance with earlier studies indicating that the TOVA distinguishes between normal children and children with ADDH[16]. The ADDH group received significantly higher scores than the controls in three of the four main TOVA variables: omission error, response time, and response time variance. Yet, when the comparison was extended to the DSM-III and DSM-IV criteria, significant differences were found only for response time and response time variance. Contrary to our hypothesis, the commission error score did not differentiate between normal and disordered children by any of the diagnostic methods. Indeed, the ADDWO children performed better than the control group on this measure. In addition, when the overall pattern of responses was considered, significant differences were found between the control and the disordered children independent of the diagnostic method. The difference was less salient when the DSM-III and DSM-IV groups were evaluated separately. A possible explanation for this finding is that the perception of attention deficit disorder as multidimensional leads to a tendency to include more children in the disordered group[20,21]. In the present study, secondary diagnoses were performed for the disordered DSM-IV groups, but not for the control group. Therefore, some of the children considered normal might have been diagnosed differently had the DSM-IV been applied[1]. These findings highlight the ability of the TOVA to demonstrate normal performance as distinct from an attention disorder. Thus, the conclusion reached with the TOVA is of high diagnostic value.

The sensitivity of the TOVA is even more impressive when the subcategories of attention deficit disorder are considered. Diagnosis according to the DSM-III showed a difference in the attention profile between the ADDH and the ADDWO groups. For the attention-maintaining features, our expectation that the ADDH group would have fewer failed responses to the target stimulus than the ADDWO group (omission error) was proved wrong. This finding, though not significant, has been reported[4] and Lovenjoy and Rasmussen[22] suggested that omission error is a characteristic of poor concentration, not of attention preservation. Thus, it is possible that in the laboratory, the ability to concentrate is more affected by the motor restlessness of the ADDH children than by the confusion of the ADDWO children. Our expectation that the time to correct response would be slower for the ADDWO than for the ADDH group was indeed confirmed, but the difference was not significant. This finding supports the earlier characterization of ADDWO children as slow and dreamy with a slow cognitive rate[4]. Likewise, our hypothesis that the response time variance would be higher in the ADDH than in the ADDWO group was proven correct, but again the difference was not significant. This finding corresponds to the notion that children with ADDH exhibit less stability in attention preservation and in ways of perceiving information. Lack of attention stability emerges from the heterogenic reactions that characterize this group[8,11].

The findings for the impulsivity feature also only partly agreed with our hypothesis. Children with ADDH are described as more impulsive than children with ADDWO[1,2,23]. The impulsivity of the former group is related to their acting before thinking, while that of the latter is related to their difficulties in completing a task[1]. Therefore, we expected the ADDH group to perform more commission errors (respond wrongly to the target stimulus) than the ADDWO group. This finding was significant only for T scores, though other scores also distinguished these groups. The ADDH group also performed worse on the postcommission response measure than the ADDWO group, but the difference was significant only for reaction time variance. With regard to the second impulsivity feature, anticipatory response (response before the stimulus appears), no differences were found between the groups, as we assumed. These results agree with other studies[16].

Similar results were noted when the subjects were diagnosed according to the DSM-IV criteria. Children with the predominantly inattentive type (ADHD-I) showed difficulties related to the attention features of the TOVA: they made more omission errors and showed slower reaction times than children with the combined type (ADHD-CT) and the predominantly hyperactive-impulsive type (ADHD-H). The ADHD-H group showed difficulties related to the hyperactivity and impulsivity features: they made more commission errors and showed greater deviation in reaction time variance than the ADHD-I group.

Finally, the ADHD-CT group made more omission errors and showed greater deviation in reaction time variance and slower reaction time than the ADHD-H group. These findings agree with our hypothesis and support the claim that children with the combined type of ADHD are the most severely disordered[20].

Other measures used in this study were the ADHD Rating Scale, the Conners Rating Scale, and the CAP, completed by parents. The validity of these questionnaires in distinguishing between normal children and children with attention deficit disorders has already been confirmed[10]. The present study showed that they are valid for use in the Israeli population. Furthermore, the questionnaires, like the TOVA, also differentiate between the attention deficit disorder groups, with the ADDWO group receiving lower scores than the ADDH group. More specifically, on the ADHD Rating Scale, although the attention factor did not distinguish the two groups, the impulsivity factor was significantly higher for the ADDH patients, and on the CAP, significant differences were found for the hyperactivity factor, with the ADDH group scoring higher. Corresponding results were noted when the subjects were diagnosed by the DSM-IV: the ADHD-I group tended to receive lower scores than the ADHD-H and the ADHD-CT groups. More specifically, the ADHD-I group achieved significantly lower scores than the other two groups in the hyperactivity and impulsivity factors. Again, the attention factor did not distinguish the groups, although the ADHD-H patients scored relatively higher on the attention factor of the CAP. It seems that this factor is also loaded with hyperactivity[4]. These findings show the power of the CAP and the ADHD Rating Scale differentiating between subcategories of attention deficit disorder and identifying the presence of hyperactivity and impulsivity symptoms, thereby contributing to the clinical picture formed by the more widespread Conners scale. Nevertheless, the ability of these questionnaires to identify attention features is poor compared to the TOVA.

Several factors have been suggested to identify the 75% of children with attention deficit disorder who respond well to stimulant medication[4,24]. Approximately 3% of all children with attention deficit disorder do not respond to any kind of medication[4]. Our study demonstrates that the TOVA can screen for responsiveness. Furthermore, as hypothesized on the basis of previous research[16], after taking methylphenidate the disordered groups showed significantly improved performance on the TOVA in almost all the factors examined. Contrary to our hypothesis, however, there were no differences in improvement among the subgroups. It is possible that the use of different doses among the various studies may have led to different results[4]. Finally, it is important to emphasize that these findings do not necessarily demonstrate that methylphenidate has positive effects on the disturbed everyday behavior of the subjects. Additional research is warranted to examine the relationship between improvement in performance on the TOVA and improvement of overt everyday behavior of children with attention deficit disorder.

This study is limited mainly by its focus on the subcategories of attention deficit disorder defined by the DSM-III, because the DSM-III-R and DSM-IV were published only after the study was already underway. Since most of the differentiated patterns found here were not significant, more extensive research is recommended. Nevertheless, the patterns match the basic accepted subcategories of attention deficit disorder. In addition, studies comparing the DSM-III and DSM-IV diagnoses have shown that the diagnosis of ADHD-I is similar to ADDWO and the diagnosis of ADHD-CT is similar to ADDH[20,21].

### CONCLUSION

Attention deficit disorder is one of the most common disorders of childhood, affecting 3% of the population[3]. Nonetheless, its diagnosis and treatment remain unclear. Our study showed that the TOVA can be applied as a valid, objective measure of the different attention profiles of subgroups of children with attention deficit disorder. This finding is of major importance to the specific clinical evaluation and treatment of affected individuals. It also waylays one of the primary concerns of parents, that is, the subjective nature of teachers' evaluations[23]. In addition, our findings demonstrate the effectiveness of methylphenidate and thereby emphasize the underlying biological mechanism of attention deficit disorder. Thus, use of the TOVA might encourage parents to both accept the diagnosis and favor medical treatment. As for the ability of the TOVA to predict responsiveness to treatment, further studies are needed to examine the correlation between improvement in performance on the TOVA and improvement in everyday behavior.

## References

1. Lahey, B.B., Pelham, W.E., Schaughency, E.A., Atkins, M.S., Marphy, H.A., Hynd, G.W., Russo, M., Hardagen, S., and Lorys-Vernon, A. (1988) Dimensions and types of attention deficit disorder with hyperactivity in children. Factor and cluster analytic approach. *J. Am. Acad. Child. Adolesc. Psychiatry* **27**, 330–335.
2. Cantwell, D.P. and Baker, L. (1992) Attention deficit disorder with or without hyperactivity: a review and comparison of matched groups. *J. Am. Acad. Child. Adolesc. Psychiatry* **31,** 432–438.
3. Barkley, R.A. (1990) ADHD clinic patent interview. In *Attention Deficit Hyperactivity Disorder: A Handbook for Diagnosis and Treatment.* Barkley, R.A., Ed. Guilford Press, New York. pp. 261–277.
4. Barkley, R.A., DuPaul, G.J., and McMurray, M.B. (1991) Attention deficit disorder with and without hyperactivity. Clinical response to three dose levels of methylphenidate. *Pediatrics* **87,** 519–531.
5. Mirsky, A.E. (1987) Behavioral and psychophysiological markers of disordered attention. *Environ. Health Perspect.* **74**, 191–199.
6. Posner, M. (1988) Structures and functions of selective attention. In *Clinical Neuropsychology and Brain Function. Research, Measurement and Practice.* Boll, A. and Bruant, B.K., Eds. American Psychological Association, Washington, D.C. pp. 169–202.
7. Lahey, B.B., Schaughency, E.A., Fram, C.L., and Strauss, C.C. (1985) Teacher rating of attention problems in children experimentally classified as exhibiting attention deficit disorder with and without hyperactivity. *J. Child Psychiatry* **24**, 613–616.
8. Bauermeister, J.J., Alegria, M., Bird, H.R., Stipec, M.R., and Canino, G. (1992) Are attentional hyperactivity deficits unidimensional or multidimensional syndromes? Empirical finding from a community survey. *J. Am. Acad. Child Adolesc. Psychiatry* **31**, 423–431.
9. Costello, A.J., Edelbrock, C.S., Kalas, R., and Dulcan, M. (1984) *The NIMH Diagnostic Interview Schedule for Children*. University of Massachusetts Medical School, Worcester.
10. Lahey, B.B. and Carlson, C.L. (1991) Validity of the diagnostic category of attention deficit disorder without hyperactivity: a review of the literature. *J. Learn. Disabil.* **24**, 110–120.
11. Lahey, B.B., Schaughency, E.A., and Strauss, L. (1984) Are attention deficit disorders with and without hyperactivity similar or dissimilar disorders? *J. Am. Acad. Child Psychiatry* **22,** 302–309.
12. King, C. and Young, R. (1982) Attention deficit with and without hyperactivity: teacher and peer perceptions. *J. Abnorm. Child Psychol*. **10**, 483–496.
13. Edelbrock, L. and Achenbach, T.M. (1984) The teacher version of the child behavior profile. I. Boys aged 6 - 11. *J. Consult. Clin. Psychol.* **52**, 207–217.
14. Frank, Y. and Ben, N. (1988) Toward a clinical subgrouping of hyperactive and nonhyperactive attention deficit disorder. Results of a comprehensive neurological and neuropsychological assessment. *J. Dis. Child* **142**, 153–155.
15. Sargent, J.A. and Scholten, C.A. (1985) On resource strategy limitation in hyperactivity. Cognitive impulsivity reconsidered. *J. Child Psychol. Psychiatry* **26**, 97–109.
16. Greenberg, L.M. and Waldman, I.D. (1993) Developmental normative data on the Test of Variables of Attention (TOVA). *J. Child Psychol. Psychiatry* **34**, 1019–1030.
17. Edelbrock, L. (1990) Child Attention Profile -CAP. In *Attention Deficit Hyperactivity Disorders: A Handbook for Diagnosis and Treatment*. Barkley, R.A., Ed. Guilford Press, New York. pp. 225–227.
18. Loney, J. and Milich, R. (1982) Hyperactivity, inattention and aggression in clinical practice. In *Advances in Developmental and Behavioral Pediatrics.* Vol. 3. Wolraich, M. and Routh, D.K., Eds. JAI Press, Greenwich, CN. pp. 113–147.
19. Goyette, C.H., Conner, C.K., and Ulrich, R.F. (1978) Normative data on the revised Conner's Parent and Teacher Rating Scale. *J. Abnorm. Child Psychol*. **6**, 221–236.
20. Morgan, A.E., Hynd, G.W., Riccio, C.A., and Hall, J. (1996) Validity of DSM-IV ADHD predominantly inattentive and combined types: relation to previous DSM diagnoses/subtype differences. *J. Am. Acad. Child Adolesc. Psychiatry* **35**, 325–333.
21. Wolraich, M.L., Hanna, J.N., Pinnock, T.Y., Baumgaentel, A., and Brown, J. (1996) Comparison of diagnostic criteria for attention-deficit hyperactivity disorder in a county-wide sample. *J. Am. Acad. Child Adolesc. Psychiatry* **35**, 319–325.
22. Lovenjoy, M.C. and Rasmussen, N.H. (1990) The validity of vigilance task in the differential diagnosis of children referred for attention and learning problems. *J. Abnorm. Child Psychol*. **18**, 671–681.
23. Teicher, M.H., Ito, Y., Glod, C.A., and Barber, N.J. (1996) Objective measurement of hyperactivity and attentional problems in ADHD. *J. Am. Acad. Child Adolesc. Psychiatry* **35**, 334–342.

24. Silva, R.R., Munoz, D.M., and Alpert, M. (1996) Carbamazepine use in children and adolescents with features of ADHD: a meta-analysis. *J. Am. Acad. Child Adolesc. Psychiatry* **35**, 352–358.

### BIOSKETCHES

**Gil Zalsman, M.D**. is a child and adolescent psychiatrist, Deputy Director of the Adolescent Inpatient Department, Geha Mental Health Center, Petah Tikva, Israel and a lecturer in psychiatry at Sackler Faculty of Medicine, Tel Aviv University, Israel. Dr. Zalsman is currently a Research Fellow at the Neuroscience Department, New York State Psychiatric Institute and Columbia University, New York. Dr. Zalsman is studying impulsive-aggression behaviors in children and adolescents and suicidal behavior and has published genetic studies in this field. zalsman@post.tau.ac.il

**Orit Pumeranz, M.D.** was a child and adolescent psychiatrist in the Child Outpatient Department, Geha Mental Health Center, Petah Tikva, Israel. She is now in a private pratice.

**Gabriel Peretz, M.A.** is a student of psychology at the Department of Psychology, Bar Ilan University, Ramat Gan, Israel.

**David H. Ben-Dor** is a child and adolescent psychiatrist, and Attending Senior Psychiatrist of the Adolescent Inpatient Department, Geha Mental Health Center, Petah Tikva, Israel. bendordh@hotmail.com

**Sharon Dekel** is a student of psychology at the Department of Psychology, Bar Ilan University, Ramat Gan, Israel.

**Neta Horesh, Ph.D.,** is a senior lecturer at the Department of Psychology, Bar Ilan University, Ramat Gan, Israel.

**Tsvi Fischel, M.D.** is Senior Psychiatrist at the Geha Mental Health Center, Petah Tikva, Israel.

**Eitan Nahshoni, M.D.** is Senior Psychiatrist at the Geha Mental Health Center, Petah Tikva, Israel. green@netvision.net.il

**Pablo H. Goldberg, M.D.** is a Child Psychiatrist working in the Department of Child Psychiatry and in the Department of Neuroscience at NYSPI/Columbia University, New York, New York.

**Jonathan Sever, M.D.** is the Director of the Child and Adolescent Outpatient Department, Geha Mental Health Center, Petah Tikva, Israel.

**Alan Apter, M.D.** is Professor of Child and Adolescent Psychiatry and the Director of the Feinberg Child Study Center, Schneider Children's Medical Center of Israel, Petah Tikva, affiliated with Sackler Faculty of Medicine, Tel Aviv University, Israel. eapter@clalit.org.il
